# Anatomical imbalance between cortical networks in autism

**DOI:** 10.1038/srep31114

**Published:** 2016-08-03

**Authors:** Takamitsu Watanabe, Geraint Rees

**Affiliations:** 1 Institute of Cognitive Neuroscience, University College London, 17 Queen Square, London, WC1N 3AR, United Kingdom; 2 Wellcome Trust Centre for Neuroimaging, University College London, 12 Queen Square, London WC1N 3BG, United Kingdom

## Abstract

Influential psychological models of autism spectrum disorder (ASD) have proposed that this prevalent developmental disorder results from impairment of global (integrative) information processing and overload of local (sensory) information. However, little neuroanatomical evidence consistent with this account has been reported. Here, we examined relative grey matter volumes (rGMVs) between three cortical networks, how they changed with age, and their relationship with core symptomatology. Using public neuroimaging data of high-functioning ASD males and age-/sex-/IQ-matched controls, we first identified age-associated atypical increases in rGMVs of the regions of two sensory systems (auditory and visual networks), and an age-related aberrant decrease in rGMV of a task-control system (fronto-parietal network, FPN) in ASD children. While the enlarged rGMV of the auditory network in ASD adults was associated with the severity of autistic socio-communicational core symptom, that of the visual network was instead correlated with the severity of restricted and repetitive behaviours in ASD. Notably, the atypically decreased rGMV of FPN predicted both of the two core symptoms. These findings suggest that disproportionate undergrowth of a task-control system (FPN) may be a common anatomical basis for the two ASD core symptoms, and relative overgrowth of the two different sensory systems selectively compounds the distinct symptoms.

Autism spectrum disorder (ASD) is characterised by socio-communicational deficits and restricted and repetitive behaviours (RRB)^1^. In prominent psychological models, these symptoms have been accounted for as the behavioural expression of a functional imbalance between global and local information processing^2^,^3^,^4^,^5^,^6^,^7^,^8^. For example, one model proposes that hyperactivity of primary sensory areas overloads higher-order cognitive processes in the autistic brains, resulting in impairment of integrative cognition and overemphasis on low-level perceptual information^2^. Weak central coherence theory^3^ and enhanced perceptual functioning theory^4^ also suggest that detail-focused and overly-enhanced lower-level perception constitutes a vital part of ASD, supported by a recent behavioural study on auditory perception^9^. In addition, underconnectivity theory and others propose that both the two core symptoms of ASD are associated with impairment of integration of global information^6^,^7^,^8^. A recent review from a Bayesian perspective has also suggested that both of autistic social deficits and RRB may be due to an imbalance between sensory precision and top-down modulation of prior belief^5^. Taken together, these psychological models suggest that the two core symptoms of ASD might be interpreted as impairment of global (integrative) information processing and overload of local (sensory) information.

However, despite many human neuroimaging studies investigating brain architectures of individuals with ASD^10^,^11^,^12^,^13^,^14^,^15^,^16^,^17^, little neuroanatomical evidence for such an account has been found. Some structural characteristics, including aberrant volumes of amygdala and superior temporal gyrus, are associated with socio-communicational deficits of ASD^18^,^19^,^20^. Other anatomical features, such as a smaller corpus callosum, are related to other non-social behaviours in ASD^21^,^22^. Although these previous findings partially support the account of ASD implied by the psychological models, direct anatomical evidence for the hypothesis has not been reported.

We searched for such neuroanatomical evidence by hypothesising that such a functional imbalance between global (integrative) and local (sensory) information processing might be reflected in an anatomical imbalance between different large-scale brain networks with distinct cognitive and perceptual functions^23^,^24^,^25^,^26^,^27^. Specifically, we set a working hypothesis that autistic core symptoms may be associated with relative anatomical immatureness of brain networks for integration of global information and reciprocal relative overgrowth of networks for lower-level perception.

To test this working hypothesis, we examined structural neuroimaging data (123 ASD and 146 age-/sex-/IQ-matched neurotypical individuals; Table 1, Supplementary Fig. 1a) that were systematically selected from MRI images shared in the Autism Brain Imaging Data Exchange (ABIDE)^28^. Using these data, we first evaluated neuroanatomical balance by calculating the ratios of grey matter volume (i.e., relative grey matter volume, rGMV) between nine representative large-scale cortical networks (Fig. 1a)^29^,^30^. We then examined age-related changes in the rGMVs of the networks, and estimated associations between the network rGMVs and severity of ASD.

## Results

### Age-related changes during childhood

As a control, we first examined the baseline of the network-based rGMVs by comparing the nine network rGMVs between ASD and age-/IQ-matched TD individuals in a relatively early part of childhood (7 ≤ age ≤ 11), and found no significant difference between the two groups (*P* > 0.05 in main effects and interactions in a repeated measures two-way ANOVA; Supplementary Fig. 2).

Next, using the entire childhood data (7 ≤ age ≤ 18), we found significant associations between age and rGMV of the regions constituting three networks (*P*_uncorrected_ < 0.05/9, *P*_Bonferroni-corrected_ < 0.05; Fig. 2). In auditory and visual networks, the rGMVs showed positive correlations with age in ASD individuals (auditory, *r* = 0.32, *P* = 0.002; visual, *r* = 0.43, *P* = 0.0002; Fig. 2a), whereas such correlations were not seen in TD group (auditory, *r* = −0.097, *P* = 0.34; visual, *r* = −0.079, *P* = 0.44; Fig. 2b). In the fronto-parietal network (FPN), rGMV was negatively correlated with age in ASD individuals (*r* = −0.34, *P* = 0.0012), whilst that was positively associated in TD children (*r* = 0.44, *P* < 10^–5^) (Fig. 2c). Moreover, the differences in correlations seen in the three networks were significant between ASD and TD groups (*z* > 2.8, *P* < 0.05). These significant age-rGMV associations were consistently observed in IQ-controlled partial correlations (Supplementary Table 2), and were robust against unspecific effects due to differences between the data collection sites (Table 2). We did not find such significant correlations in the other six networks.

These findings suggest that, in our high-functioning ASD population, rGMVs of auditory and visual networks atypically increase during childhood, and that of FPN aberrantly decreases. In fact, we confirmed that such atypical anatomical development during childhood resulted in significant differences in network rGMVs between ASD and TD adults (18 < age ≤ 40) (*F*_(8,746)_ = 325.5, *P* < 0.0001 as interactions in a repeated measure two-way ANOVA, *P*_Bonferroni-corrected_ < 0.05 in post-hoc two-sample *t*-tests; Fig. 3). The rGMVs of auditory and visual networks were significantly larger in adult ASD group than in adult TD group (auditory, *t*_(82)_ = 3.8, Cohen’s *d* = 0.81, *P* = 0.0003; visual, *t*_(82)_ = 3.6, *d* = 0.75, *P* = 0.0004 in two-sample *t*-tests), whereas that of FPN was significantly smaller in ASD (*t*_(82)_ = 5.6, *d* = 0.89, *P* < 0.0001).

### Associations with ASD symptoms

Using the adult ASD data, we then examined whether these atypical anatomical developments were associated with severity of the core symptoms of ASD (Fig. 4). The disproportionate overgrowth of rGMV of auditory network was correlated with severity of autistic socio-communicational deficits (*r* = 0.51, *P* = 0.002; Fig. 4a), whereas that of visual network was related with the extent of autistic RRB (*r* = 0.56, *P* = 0.0004; Fig. 4b). These correlations were specific to each core symptom (*z* > 2.0, *P* < 0.05). In contrast, the atypical undergrowth of rGMV of FPN was correlated with both of the two core symptoms of ASD (socio-communicational, *r* = −0.48, *P* = 0.003; RRB, *r* = −0.46, *P* = 0.005; Fig. 4c).

These symptom-rGMV associations were consistently observed after effects of IQ and age were controlled (Supplementary Table 3) or even when they were calculated using different parts of the datasets (Table 3). In addition, the associations between severity of social symptoms and rGMV were qualitatively reproduced when the correlations were separately calculated for ADIR-social and ADIR-communication scores (Supplementary Fig. 3, (Supplementary Table 4). We did not find such significant symptom-rGMV correlations in the other networks.

Furthermore, this anatomical imbalance between different networks and its relationship with autistic symptoms were consistently observed when such anatomical balance was quantified based on cortical thickness rather than GMV (Supplementary Fig. 4; Supplementary Methods).

In contrast, we could not find such significant rGMV-symptom correlations in data obtained from autistic children (*P* > 0.15). It is possibly because, as shown above (Fig. 2), ongoing developmental brain changes during childhood may act as a confounding factor and make it difficult to detect such neuroanatomy-symptom associations in the childhood dataset.

### Regions responsible for symptom-rGMV associations

Finally, we searched for brain regions responsible for the significant correlations between rGMVs and ASD symptoms seen in the three brain networks (Fig. 5a). Regarding the associations with severity of socio-communicational deficits, rGMVs of medial prefrontal cortex (mPFC) in FPN and bilateral posterior insulae in auditory network were significantly related to such impairment (mPFC, *r* = −0.61, *P* = 0.00009; right posterior insula, *r* = 0.57, *P* = 0.0003; left posterior insula, *r* = 0.47, *P* = 0.004; *P*_FDR_ < 0.05; Fig. 5b). Regarding RRB, rGMVs of ventro-/dorso- lateral prefrontal cortex (VLPFC/DLFC) in FPN and three lateral occipital regions in visual network were associated with ADIR-RRB (*r* > 0.40, *P* ≤ 0.01; *P*_FDR_ < 0.05; Fig. 5c). Such significant rGMV-symptom correlations were not found in the other regions. As discussed below, these significant associations found in specific brain regions were consistent with previous literature on autism and relevant cognitive functions.

## Discussion

The current study has compared the anatomical balance among the nine cortical networks for high-functioning ASD and age-/sex-/IQ-matched TD groups, and found atypical age-related increases in rGMVs of auditory/visual networks and a decrease in rGMV of FPN in ASD children. In ASD adults, the increased rGMV of auditory network was specifically correlated with autistic socio-communicational deficit, whereas that of visual network was selectively associated with RRB. The disproportionate low rGMV of FPN was predictive of both of the two ASD core symptoms. These findings suggest that the relative undergrowth of FPN is an anatomical basis for the two autistic core symptoms, whereas disproportionate overgrowth of the two different sensory networks allows the diversity of these symptoms.

### Network-based interpretation

According to network-based perspectives on functional and anatomical brain architectures^23^,^24^,^25^,^26^,^27^, the auditory and visual networks are mainly involved with lower-level perception^29^, whereas FPN supposedly controls attention, integrates the information processed in the other networks, and plays a central role in various cognitive functions^30^,^31^,^32^. From such a viewpoint, if we assume a positive correlation between regional GMV and cognitive functions relevant to the regions^33^, the current results are consistent with prominent theories of autism^2^,^3^,^4^,^5^,^6^,^7^,^8^, and appear to support their collective hypothesis that ASD symptoms are interpreted as a behavioural expression of impairment of information integration and enhancement of low-level perceptual information processing.

### Region-based interpretation

Specific brain regions were largely responsible for these significant associations between network rGMVs and symptom severity (Fig. 5).

For socio-communication deficits, the correlation with rGMV of FPN appeared to largely depend on atypical anatomical undergrowth of mPFC (Fig. 5b). Such a region is critically involved in various social cognition paradigms in neurotypical individuals^34^ and decreased mPFC activity is observed in ASD populations^35^,^36^,^37^,^38^. In the auditory network, grey matter in bilateral posterior insulae was correlated with socio-communication deficits (Fig. 5b). Such increases in GMV in posterior insulae are associated with increased sensitivity to pain and tactile stimulation in neurotypical individuals^39^,^40^; and such changes can be correlated with social deficits in autism^41^. Therefore, it is reasonable to suggest that the relative overgrowth of posterior insulae might induce hypersensitivity in autism and compound socio-communicational deficits of ASD.

As for RRB, the relative undergrowth of DLPFC and VLPFC was responsible brain regions in FPN (Fig. 5c), which is consistent with previously reported associations between lateral prefrontal activity and performance of cognitive control in TD individuals^42^ and ASD group^43^.

In the visual network, disproportionately overgrown bilateral extra-striate occipital regions are observed in autistic individuals (Fig. 5c), which is consistent with a recent behavioural study showing a significant relationship between atypical enhancement of visual perception and narrowed attention in autism^44^. In addition, this symptom-rGMV association is also congruent with recent neuroimaging studies indicating that the hyper-activations of the lateral primary visual regions could reduce attention alteration in TD individuals^45^ and ASD group^46^.

### Consistency with anatomical studies

It is difficult to directly compare the current findings with previous findings on GMV of autistic brains, because most of this literature directly examined GMV without calculating ratios of GMV between different regions or different networks^10^,^11^,^12^,^13^. However, the current observations are consistent with some previous reports and meta-analytic results. For example, the atypically increased rGMV of the visual network that we observed in the ASD group is in accordance with a meta-analysis showing aberrant GMV increases of lateral occipital regions^17^. The age-related overgrowth of the visual network is also consistent with another meta-analysis^47^. In addition, this meta-analysis has also reported age-associated GMV decreases of parietal regions, which is congruent with the age-related rGMV decrease in FPN in the current study. Moreover, a recent longitudinal study has reported that frontal and parietal areas showed sharper age-related decreases in GMV than occipital regions^48^, which is congruent with the dissociation of age-associated rGMV changes between FPN and visual network in this study.

### Limitations

One of the main limitations of the current study is the cross-sectional study design using data collected from multiple sites. In theory, compared with estimation of GMV, calculation of rGMV is more robust against noise induced by the individual variability and difference across imaging sites. In addition, the main observations were qualitatively reproduced even when we calculated them using different parts of the datasets (Tables 2 and 3). However, ideally, developmental changes in brain anatomy should be investigated in a longitudinal design using the quality-controlled same MRI scanner^10^,^13^.

Another limitation is the lack of consideration of any specific effects of puberty. Puberty has significant impacts on brain anatomy^15^,^17^,^49^,^50^, but the current observations did not consider these effects because the ABIDE database did not include such information. For the same technical reason, we could not investigate changes in network-based anatomical balance during toddlerhood and early childhood before the age of seven.

We need to be careful when interpreting and potentially generalising the current observations. First, the current findings do not directly indicate an imbalance of absolute values of network GMV in autistic brains. The rGMV used in the current analysis is an abstract index used to estimate neuroanatomical balance between networks, and therefore it can be different from the absolute values of GMV. Second, the current findings could be affected by the way in which we defined the regions of interest (ROIs). Specifically, the ROI coordinates and network classifications were based on previous functional neuroimaging studies of TD individuals^29^,^30^, and so may not reflect the ASD population. Finally, we cannot conclude that the structural imbalance between the three networks we observed is an underlying cause of ASD. As it is possible to reliably diagnose autism in two-year-old children^51^, our findings concerning older ASD individuals (i.e., ≥7 years old) might not represent a primary pathological mechanism of this developmental disorder but instead reflect compensatory neuroanatomical processes emerging during childhood and adolescence in individuals with ASD.

## Conclusion

Using publicly shared neuroimaging datasets, the current study has examined anatomical balance between large-scale brain networks in autism and matched controls. Consequently, we have found atypical relative overgrowth of brain regions in FPN and disproportionate undergrowth of auditory and visual networks. Moreover, this relative undergrowth of the two different sensory systems is selectively correlated with two different core symptoms of autism, and this relative overgrowth of FPN is related to both of the autistic behaviours. These findings provide empirical evidence for several prominent autism theories, and support for a network-based framework of unified understanding of seemingly diverse ASD symptoms.

## Materials and Methods

### Data

Anatomical data were selected from T1-weighted MRI images of 468 autistic individuals who were diagnosed as ASD based on DSM-IV-TR and 560 TD controls that were recorded in 3.0T MRI scanners in multiple institutes, and now are shared in ABIDE^28^. According to the data repository, the data collection was approved by the corresponding local Institutional Review Boards, and was performed in accordance with the corresponding institutional regulations (i.e., Review Boards and their regulations in New York University, Pittsburgh University, San Diego State University, University California Los Angeles, California Institute of Technology, and Trinity Centre for Health Sciences). Written informed consents were obtained from all the participants. The data were fully anonymised before being publicly shared. The imaging protocols are considered to be equivalent across different institutes (T1-weighted protocol; TR ≃ 2.5 s, TE ≃ 3 ms, thickness ≃1.1 mm).

First, we systematically selected MRI images that were recorded from right-handed high-functioning ASD males who had average or above-average intelligence (full IQ ≥ 80) evaluated by WISC, WASI, or WAIS, and were given Autism Diagnostic Interview-Revised (ADIR)^52^ scores. Individuals who had medication history of anti-psychotic drugs or took any medication on the scanning day were also excluded (Supplementary Fig. 1). The right handedness was determined based on the demographic data sheet accompanied by the MRI dataset (i.e., ‘R’ in Handedness Category or positive number in Handedness Scores). Second, we classified the participants into childhood group (age ≤ 18) and adult group (18 < age), and obtained data of 89 ASD children (7 ≤ age ≤ 18) and those of 34 ASD adults (18 < age ≤ 40). Finally, we selected data of TD individuals by matching their sex, IQ, age, handedness, and institutes collecting data, and gathered data of 96 TD children and those of 50 TD adults.

There was no significant difference in any of age, IQ, and distribution of recording sites between ASD and TD groups (Table 1). Moreover, no significant difference in age and IQ was seen between the data collection sites (*P* ≥ 0.1 in one-way analysis of variance; Supplementary Table 1).

We used ADIR scores rather than Autism Diagnostic Observations Schedule (ADOS) scores, because the number of ASD adults scored by ADIR (*N* = 123) was larger than that of ADOS (*N* = 82). For the same reason, the current study employed only male data (ASD, *N* = 123) rather than female data (ASD, *N* = 15).

### Data processing

These anatomical MRI images were preprocessed for the following GMV analysis in SPM12^53^. They were segmented into grey matter, white matter, and cerebrospinal fluid in the native space using the New Segment Toolbox^54^. The segmented grey matter images underwent alignment, warp to a template space, resampling down to 1.5 mm isotropic voxels, and registration to a participant-specific template with the DARTEL Toolbox^55^. Using deformation parameters estimated by the DARTEL toolbox, the grey matter images were normalised to MNI spaces and smoothed with a Gaussian kernel (FWHM = 8 mm). Because this preprocessing procedures in DARTEL Toolbox included a so-called modulation process to preserve the volume of a particular tissue within a voxel (see Section 25.5.3 in SPM12 manual in www.fil.ion.ucl.ac.uk/spm/doc/manual.pdf), signal values in the resultant preprocessed images were supposed to represent GMV in the voxels. The images were then normalised by being divided by the whole-brain GMV. This normalisation was expected to control for not only individual differences in whole brain volumes but also unspecific differences between the data collection sites.

**Figure 1:**
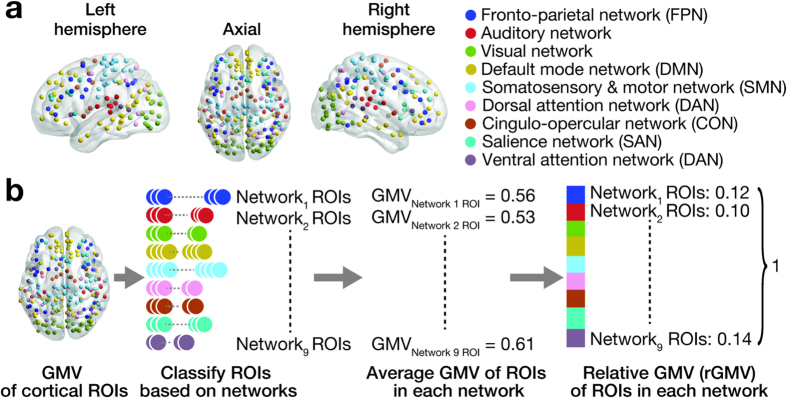
(**a**) Nine large-scale brain networks. 213 cortical ROIs were defined in previous study^29^,^30^, and are categorised into nine brain networks. No ROI was shared between different networks. (**b)** Analysis procedures. We first extracted grey matter volume (GMV) for each ROI from preprocessed anatomical neuroimaging data, and then classified the ROIs to the nine networks (panel a). After calculating average GMV for each network, we normalised the GMVs and estimated relative GMVs (rGMVs) for the networks. This procedure was repeated for each participant.

**Figure 2:**
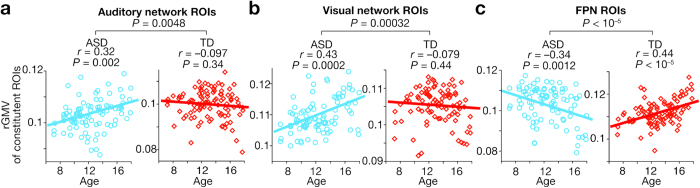
Age-associated rGMV changes. Using the childhood data (age ≤ 18), we found significant correlations between age and rGMV of regions in three of the nine brain networks (*P*_Bonferroni-corrected_ < 0.05). The rGMVs of auditory and visual networks showed significantly positive correlations with age in ASD children, but not in neurotypical ones (panels a,b). In contrast, the rGMV of FPN was negatively correlated with age in autism, and was positively correlated in neurotypical population (panel c). These correlations were qualitatively preserved after controlling the effects of IQ (Supplementary Table 2) or even when considering effects of recording sites (Table 2).

**Figure 3:**
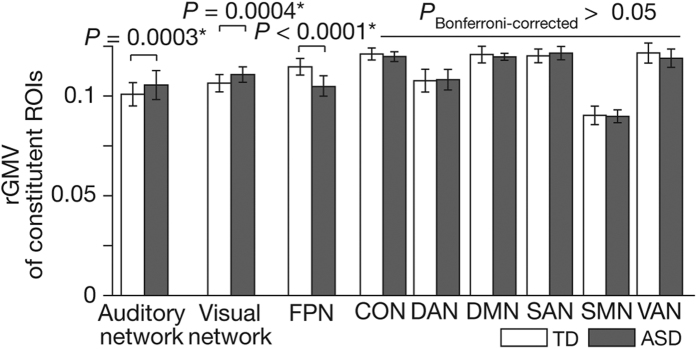
Comparison of rGMVs between brain networks. We compared rGMVs between the nine brain networks. In the adult data (18 < age ≤ 40), rGMVs of auditory and visual networks in autism were significantly larger than those in the neurotypical adults, whereas that of FPN was smaller in autism (*F*_(8,746)_ = 325.5, *P* < 0.0001 as interactions in a repeated measure two-way ANOVA; *P_Bonferroni-corrected_ < 0.05 in post-hoc two-sample *t*-tests). Error bars: std. The abbreviations of network names were spelled out in Fig. 1a.

**Figure 4:**
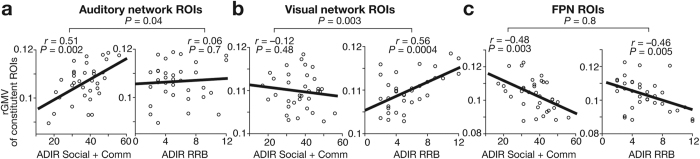
Correlations between rGMVs and ASD symptoms. Using data of ASD adults, we estimated associations between rGMVs and severity of autism, which was clinically evaluated with ADIR^52^. Individuals with larger rGMVs of auditory network were likely to have severer deficits in socio-communicational interactions (ADIR Social + Communication) (panel a), whereas those with larger rGMVs of visual network tended to show severer RRB (ADIR-RRB) (panel b) (*P*_Bonferroni-corrected_ < 0.05). Autistic individuals with smaller rGMVs of FPN had severer symptoms in both core symptoms (panel c).

**Figure 5:**
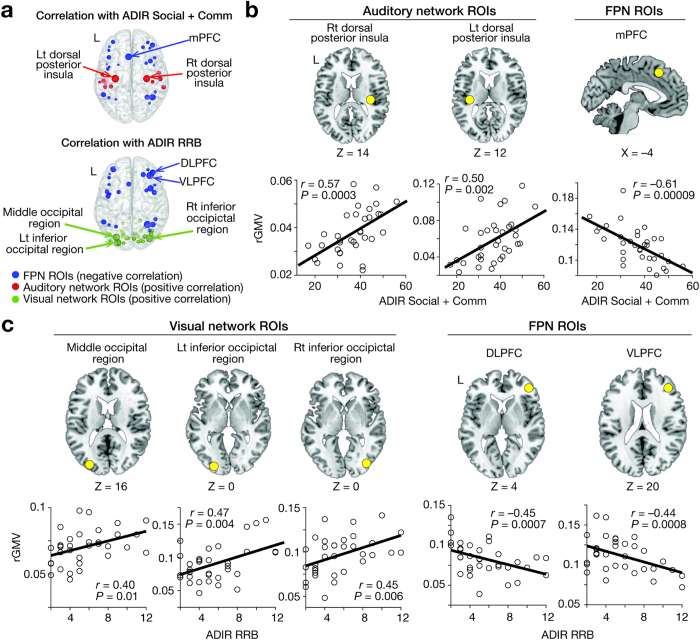
(**a**) Using data of ASD adults, we searched ROIs in auditory, visual, and fronto-parietal networks for focal regions whose rGMVs were significantly correlated with ASD severity. The sizes of the circles represent the magnitudes of the symptom-rGMV correlations. (**b**) In auditory network, rGMVs of bilateral dorsal posterior insulae had significant positive correlations with socio-communicational deficits, whereas rGMV of medial prefrontal cortex (mPFC) in FPN showed a negative correlation. (**c**) In visual network, rGMVs of three lateral occipital regions in visual network were positively correlated with RRB, and those of two dorso-/ventro-lateral prefrontal cortical regions (DLPFC/VLPFC) in FPN showed negative correlations.

For each image, we extracted GMVs of 213 cortical ROIs, which were defined as 4 mm-radius spheres whose centre coordinates were determined in previous functional neuroimaging studies^29^,^30^. To focus on cortical networks, we excluded 51 ROIs with ‘subcortical’ or ‘uncertain’ labels from the original 264 ROIs^29^,^30^. The GMV of each ROI was determined as the average GMV of the corresponding 4 mm-raidus sphere. Because the minimum distance between the 213 ROIs was 10.0 mm, we set the radius of the ROI sphere at 4 mm and avoided overlap between different ROI areas. We then classified the ROIs into the nine cortical networks^29^,^30^ (Fig. 1a,b), calculated a mean GMV for each network by averaging GMVs across the ROIs constituting each network, and estimated a relative GMV (rGMV) for each network by normalising the mean GMVs among the networks. The rGMVs are thus considered to represent anatomical balance between the nine cortical networks for each participant. Note that no ROI was shared between different networks.

This ROI classification was indirectly justified through calculating inter-volumetric correlations between GMVs of the ROIs as follows: for example, in TD adult group, for each network (e.g., FPN), we first averaged the across-participant GMV correlations between the ROIs belonging to the specific network (here, FPN). In the meantime, we also estimated the average of the GMV correlations between the ROIs in the network (i.e., ROIs in FPN) and ROIs belonging to the other eight networks (ROIs in the networks rather than FPN). We repeated this procedure for every network in every participant group. As a result, we confirmed that, for all the networks in all the participant groups, the within-network inter-volumetric correlations were larger than the between-network correlations. This contrast was also supported by statistical tests (ASD children: *t*_8_ = 3.4, *P*_uncorrected_ = 0.008; ASD adults: *t*_8_ = 4.0, *P*_uncorrected_ = 0.003; TD children: *t*_8_ = 4.3, *P*_uncorrected_ = 0.002; TD adults: *t*_8_ = 3.6, *P*_uncorrected_ = 0.007 in paired *t*-tests; all *P*_FDR_ < 0.05).

The current study focused on the brain regions in the network (i.e., nodes) but not on the connections between them (i.e., edges), because the dataset did not contain neuroimaging data to evaluate anatomical connections in individual brains.

### Associations with age during childhood

As a baseline, we first compared rGMVs of a relatively early part of the childhood data (7 ≤ age ≤ 11) between ASD and TD groups with a repeated measures two-way ANOVA of rGMV (two types of group [ASD/TD] × nine types of network). This age threshold (i.e., age of 11) was chosen to reduce effects of puberty with keeping the sample size. Demographic properties were controlled between the 34 ASD and 12 TD children (age, IQ: *P* > 0.15 in two-sample *t*-tests).

Next, using the entire childhood data (7 ≤ age ≤ 18), we calculated Pearson’s correlation coefficients between age and network rGMVs, and compared them between ASD and TD children. To minimise effects of IQ^16^,^56^,^57^, we also estimated partial correlations controlled by Full, Verbal, and Performance IQ. The statistical significance was adjusted by Bonferroni correction for multiple comparisons across the nine networks (α = 0.05/9).

To evaluate unspecific effects of differences in data collection sites on the observed associations, we repeatedly calculated the age-rGMV correlations using different subsets of the data excluding datasets collected in different institutes.

### Associations with symptoms in adults

Using the adult data, we first compared rGMVs between ASD and TD groups with a repeated measures two-way ANOVA (two types of group [ASD/TD] × nine types of network) and post-hoc two-sample t-tests adjusted by Bonferroni correction. We then calculated correlation coefficients between the network rGMVs and individual severity of ASD. The ASD severity was evaluated as ADIR social, communication, and repetitive/restricted behaviours (RRB). According to the latest diagnosis criteria of ASD in DSM-5^58^, scores for autistic social deficit and communicational deficit were merged into one index by summing ADIR-social and ADIR-communication. We also calculated partial correlations controlled by age and IQ. The difference in the correlations was examined between the adult ASD and TD groups. By calculating the correlations using different parts of the datasets, we also evaluated the robustness of the rGMV-symptom associations against unspecific effects of the differences in data collection sites.

### ROI-based associations with symptoms

Finally, we searched for ROIs responsible for the significant associations between network rGMVs and ASD severity. The rGMV for an ROI belonging to a certain network was calculated as the ratio of the GMV of the ROI to the summation of average GMVs of all the other networks as follows: (rGMV of ROI_*i*_ in Network_*j*_) = (GMV of ROI_*i*_)/∑_*k≠j*_(average GMV of Network_*k*_). We then estimated correlation coefficients between the ROI rGMVs and ASD severity, and searched for ROIs whose correlations were statistically significant. The significance was corrected for multiple comparisons across the ROIs included in the corresponding network by setting false discovery rate (FDR) at 0.05. We used FDR-based correction because the application of Bonferroni correction to this case, which consisted of ≥12 comparisons, was too conservative and likely to increase false negative.

## Additional Information

**How to cite this article**: Watanabe, T. and Rees, G. Anatomical imbalance between cortical networks in autism. *Sci. Rep.* **6**, 31114; doi: 10.1038/srep31114 (2016).

## Supplementary Material

Supplementary Information

## Figures and Tables

**Table 1:** Demographic data.

	ASD	TD	*P* value
Children (<18 yo)			
*N*	89	96	
Sex	Male	Male	
Handedness	Right	Right	
Age	12.4 ± 3.0	13.1 ± 2.6	0.1
FIQ	106.4 ± 14.4	108.1 ± 10.7	0.3
VIQ	105.0 ± 12.8	107.6 ± 11.2	0.2
PIQ	106.9 ± 16.4	106.7 ± 11.5	0.9
Distribution of recording sites	—	—	0.14
ADIR Social	19.3 ± 5.2	—	
ADIR Communication	15.7 ± 4.3	—	
ADIR RRB	6.1 ± 2.5	—	
Adults (18 yo≤)			
*N*	34	50	
Sex	Male	Male	
Handedness	Right	Right	
Age	23.9 ± 5.5	24.0 ± 5.0	0.9
FIQ	111.9 ± 15.0	116.18 ± 9.1	0.2
VIQ	109.7 ± 16.4	115.3 ± 10.9	0.2
PIQ	110.8 ± 14.2	113.5 ± 8.7	0.4
Distribution of recording sites	—	—	0.29
ADIR Social	20.1 ± 5.4	—	
ADIR Communication	16.8 ± 4.6	—	
ADIR RRB	5.6 ± 2.9	—	

FIQ/VIQ/PIQ, full-scale/verbal/performance IQ; ADIR, Autism Diagnostic Interview-Revised^52^; RRB, repetitive and restricted behaviour. Mean ± std.

**Table 2:** Age-rGMV correlations calculated after excluding data collected in a specific site.

	ASD children			TD children		
	Auditory Network	Visual Network	FPN	Auditory Network	Visual Network	FPN
without PITT data	0.34*	0.44*	−0.34*	−0.11	−0.09	0.44*
without SDSU data	0.32*	0.45*	−0.33*	−0.10	−0.12	0.44*
without UCLA data	0.44*	0.36*	−0.56*	−0.12	−0.09	0.44*
without NYU data	0.30*	0.30*	−0.32*	−0.11	−0.13	0.34*

*P < 0.05. PITT, Pittsburgh, University of Pittsburgh School of Medicine; SDSU, San Diego State University; UCLA, University of California Los Angels; NYU, New York University Langdon Medical Centre.

**Table 3:** Symptom-rGMV correlations calculated after excluding data collected in a specific site.

	Correlation with socio-communicational deficits			Correlation with repetitive restricted behaviours.		
	Auditory Network	Visual Network	FPN	Auditory Network	Visual Network	FPN
without CALTECH data	0.49*	−0.12	−0.46*	0.009	0.73*	−0.54*
without TRINITY data	0.53*	−0.08	−0.55*	0.09	0.51*	−0.43*
without PITT data	0.51*	−0.10	−0.40*	−0.008	0.49*	−0.44*
without NYU data	0.5*	−0.03	−0.49*	0.13	0.47*	−0.44*

*P < 0.05. CALTECH, California Institute of Technology; TRINITY, Trinity Centre for Health Sciences; PITT, Pittsburgh, University of Pittsburgh School of Medicine; NYU, New York University Langdon Medical Centre.

## References

[b1] Lai M.-C., Lombardo M. V. & Baron-Cohen S. Autism. Lancet 383, 896–910 (2014). 24074734 10.1016/S0140-6736(13)61539-1

[b2] Belmonte M. K. *et al.* Autism as a disorder of neural information processing: directions for research and targets for therapy. Mol. Psychiatry 9, 646–663 (2004). 15037868 10.1038/sj.mp.4001499

[b3] Happé F. & Frith U. The weak coherence account: detail-focused cognitive style in autism spectrum disorders. J Autism Dev Disord 36, 5–25 (2006). 16450045 10.1007/s10803-005-0039-0

[b4] Mottron L., Dawson M., Soulières I., Hubert B. & Burack J. Enhanced Perceptual Functioning in Autism: An Update, and Eight Principles of Autistic Perception. J Autism Dev Disord 36, 27–43 (2006). 16453071 10.1007/s10803-005-0040-7

[b5] Lawson R. P., Rees G. & Friston K. J. An aberrant precision account of autism. Front Hum Neurosci 8, 302 (2014). 24860482 10.3389/fnhum.2014.00302 PMC4030191

[b6] Williams D. L., Goldstein G. & Minshew N. J. Neuropsychologic functioning in children with autism: further evidence for disordered complex information-processing. Child Neuropsychol 12, 279–298 (2006). 16911973 10.1080/09297040600681190 PMC1803025

[b7] Kana R. K., Libero L. E. & Moore M. S. Disrupted cortical connectivity theory as an explanatory model for autism spectrum disorders. Physics of Life Reviews 8, 410–437 (2011). 22018722 10.1016/j.plrev.2011.10.001

[b8] Just M. A., Keller T. A., Malave V. L., Kana R. K. & Varma S. Autism as a neural systems disorder: a theory of frontal-posterior underconnectivity. Neuroscience & Biobehavioral Reviews 36, 1292–1313 (2012). 22353426 10.1016/j.neubiorev.2012.02.007 PMC3341852

[b9] Lawson R. P., Aylward J., White S. & Rees G. A striking reduction of simple loudness adaptation in autism. Sci Rep 5, 16157 (2015). 26537694 10.1038/srep16157 PMC4633623

[b10] Amaral D. G., Schumann C. M. & Nordahl C. W. Neuroanatomy of autism. Trends Neurosci. 31, 137–145 (2008). 18258309 10.1016/j.tins.2007.12.005

[b11] Courchesne E. *et al.* Mapping early brain development in autism. Neuron 56, 399–413 (2007). 17964254 10.1016/j.neuron.2007.10.016

[b12] Ecker C., Bookheimer S. Y. & Murphy D. G. M. Neuroimaging in autism spectrum disorder: brain structure and function across the lifespan. Lancet Neurol 14, 1121–1134 (2015). 25891007 10.1016/S1474-4422(15)00050-2

[b13] Lainhart J. E. Brain imaging research in autism spectrum disorders: in search of neuropathology and health across the lifespan. Curr Opin Psychiatry 28, 76–82 (2015). 25602243 10.1097/YCO.0000000000000130 PMC4465432

[b14] Haar S., Berman S., Behrmann M. & Dinstein I. Anatomical Abnormalities in Autism? Cereb. Cortex (2014). 10.1093/cercor/bhu242. PMC13377743 25316335

[b15] Hernandez L. M., Rudie J. D., Green S. A., Bookheimer S. & Dapretto M. Neural signatures of autism spectrum disorders: insights into brain network dynamics. Neuropsychopharmacology 40, 171–189 (2015). 25011468 10.1038/npp.2014.172 PMC4262896

[b16] Stanfield A. C. *et al.* Towards a neuroanatomy of autism: a systematic review and meta-analysis of structural magnetic resonance imaging studies. Eur. Psychiatry 23, 289–299 (2008). 17765485 10.1016/j.eurpsy.2007.05.006

[b17] Nickl-Jockschat T. *et al.* Brain structure anomalies in autism spectrum disorder–a meta-analysis of VBM studies using anatomic likelihood estimation. Hum Brain Mapp 33, 1470–1489 (2012). 21692142 10.1002/hbm.21299 PMC4801488

[b18] Munson J. *et al.* Amygdalar volume and behavioral development in autism. Arch. Gen. Psychiatry 63, 686–693 (2006). 16754842 10.1001/archpsyc.63.6.686

[b19] Bigler E. D. *et al.* Superior temporal gyrus, language function, and autism. Dev Neuropsychol 31, 217–238 (2007). 17488217 10.1080/87565640701190841

[b20] Schumann C. M., Barnes C. C., Lord C. & Courchesne E. Amygdala enlargement in toddlers with autism related to severity of social and communication impairments. Biol. Psychiatry 66, 942–949 (2009). 19726029 10.1016/j.biopsych.2009.07.007 PMC2795360

[b21] Just M. A., Cherkassky V. L., Keller T. A., Kana R. K. & Minshew N. J. Functional and anatomical cortical underconnectivity in autism: evidence from an FMRI study of an executive function task and corpus callosum morphometry. Cereb. Cortex 17, 951–961 (2007). 16772313 10.1093/cercor/bhl006 PMC4500121

[b22] Zielinski B. A. *et al.* scMRI reveals large-scale brain network abnormalities in autism. PLos One 7, e49172 (2012). 23185305 10.1371/journal.pone.0049172 PMC3504046

[b23] Craddock R. C. *et al.* Imaging human connectomes at the macroscale. Nat. Methods 10, 524–539 (2013). 23722212 10.1038/nmeth.2482 PMC4096321

[b24] van den Heuvel M. P. & Sporns O. Network hubs in the human brain. Trends Cogn. Sci. (Regul. Ed.) 17, 683–696 (2013). 24231140 10.1016/j.tics.2013.09.012

[b25] Medaglia J. D., Lynall M.-E. & Bassett D. S. Cognitive network neuroscience. J Cogn Neurosci 27, 1471–1491 (2015). 25803596 10.1162/jocn_a_00810 PMC4854276

[b26] Vértes P. E. & Bullmore E. T. Annual Research Review: Growth connectomics - the organization and reorganization of brain networks during normal and abnormal development. J Child Psychol Psychiatry, 10.1111/jcpp.12365 (2014). PMC4359009 25441756

[b27] Fair D. A. & Schlaggar B. L. The development of human functional brain networks. Neuron 67, 735–748 (2010). 20826306 10.1016/j.neuron.2010.08.017 PMC2941973

[b28] Di Martino A. *et al.* The autism brain imaging data exchange: towards a large-scale evaluation of the intrinsic brain architecture in autism. Mol. Psychiatry 19, 659–667 (2014). 23774715 10.1038/mp.2013.78 PMC4162310

[b29] Power J. D. *et al.* Functional network organization of the human brain. Neuron 72, 665–678 (2011). 22099467 10.1016/j.neuron.2011.09.006 PMC3222858

[b30] Cole M. W. *et al.* Multi-task connectivity reveals flexible hubs for adaptive task control. Nat. Neurosci. 16, 1348–1355 (2013). 23892552 10.1038/nn.3470 PMC3758404

[b31] Chadick J. Z. & Gazzaley A. Differential coupling of visual cortex with default or frontal-parietal network based on goals. Nat. Neurosci. 14, 830–832 (2011). 21623362 10.1038/nn.2823 PMC3125492

[b32] Zanto T. P. & Gazzaley A. Fronto-parietal network: flexible hub of cognitive control. Trends Cogn. Sci. (Regul. Ed.) 17, 602–603 (2013). 24129332 10.1016/j.tics.2013.10.001 PMC3873155

[b33] Kanai R. & Rees G. The structural basis of inter-individual differences in human behaviour and cognition. Nat. Rev. Neurosci. 12, 231–242 (2011). 21407245 10.1038/nrn3000

[b34] Frith C. D. & Frith U. Mechanisms of social cognition. Annu Rev Psychol 63, 287–313 (2012). 21838544 10.1146/annurev-psych-120710-100449

[b35] Kaiser M. D. & Pelphrey K. A. Disrupted action perception in autism: behavioral evidence, neuroendophenotypes, and diagnostic utility. Dev Cogn Neurosci 2, 25–35 (2012). 22682727 10.1016/j.dcn.2011.05.005 PMC6987680

[b36] Kennedy D. P. & Adolphs R. The social brain in psychiatric and neurological disorders. Trends Cogn. Sci. (Regul. Ed.) 16, 559–572 (2012). 23047070 10.1016/j.tics.2012.09.006 PMC3606817

[b37] Meyer-Lindenberg A. & Tost H. Neural mechanisms of social risk for psychiatric disorders. Nat. Neurosci. 15, 663–668 (2012). 22504349 10.1038/nn.3083

[b38] Watanabe T. *et al.* Diminished medial prefrontal activity behind autistic social judgments of incongruent information. PLos One 7, e39561 (2012). 22745788 10.1371/journal.pone.0039561 PMC3382122

[b39] Singer T., Critchley H. D. & Preuschoff K. A common role of insula in feelings, empathy and uncertainty. Trends Cogn. Sci. (Regul. Ed.) 13, 334–340 (2009). 19643659 10.1016/j.tics.2009.05.001

[b40] Segerdahl A. R., Mezue M., Okell T. W., Farrar J. T. & Tracey I. The dorsal posterior insula subserves a fundamental role in human pain. Nat. Neurosci. 18, 499–500 (2015). 25751532 10.1038/nn.3969 PMC6783299

[b41] Robertson A. E. & Simmons D. R. The relationship between sensory sensitivity and autistic traits in the general population. J Autism Dev Disord 43, 775–784 (2013). 22832890 10.1007/s10803-012-1608-7

[b42] Aron A. R., Robbins T. W. & Poldrack R. A. Inhibitrion and the right inferior frontal cortex: one decade on. Trends Cogn. Sci. (Regul. Ed.) 18, 177–185 (2014). 24440116 10.1016/j.tics.2013.12.003

[b43] Keehn B., Müller R.-A. & Townsend J. Atypical attentional networks and the emergence of autism. Neuroscience & Biobehavioral Reviews 37, 164–183 (2013). 23206665 10.1016/j.neubiorev.2012.11.014 PMC3563720

[b44] Robertson C. E., Kravitz D. J., Freyberg J., Baron-Cohen S. & Baker C. I. Slower rate of binocular rivalry in autism. J. Neurosci. 33, 16983–16991 (2013). 24155303 10.1523/JNEUROSCI.0448-13.2013 PMC3807027

[b45] Watanabe T., Masuda N., Megumi F., Kanai R. & Rees G. Energy landscape and dynamics of brain activity during human bistable perception. Nat Commun 5, 4765 (2014). 25163855 10.1038/ncomms5765 PMC4174295

[b46] Robertson C. E. *et al.* Global motion perception deficits in autism are reflected as early as primary visual cortex. Brain 137, 2588–2599 (2014). 25060095 10.1093/brain/awu189 PMC4132651

[b47] DeRamus T. P. & Kana R. K. Anatomical likelihood estimation meta-analysis of grey and white matter anomalies in autism spectrum disorders. Neuroimage Clin 7, 525–536 (2015). 25844306 10.1016/j.nicl.2014.11.004 PMC4375647

[b48] Lange N. *et al.* Longitudinal volumetric brain changes in autism spectrum disorder ages 6–35 years. Autism Res 8, 82–93 (2015). 25381736 10.1002/aur.1427 PMC4344386

[b49] Blakemore S.-J. Development of the social brain in adolescence. J R Soc Med 105, 111–116 (2012). 22434810 10.1258/jrsm.2011.110221 PMC3308644

[b50] Uddin L. Q., Supekar K. & Menon V. Reconceptualizing functional brain connectivity in autism from a developmental perspective. Front Hum Neurosci 7, 458 (2013). 23966925 10.3389/fnhum.2013.00458 PMC3735986

[b51] Lord C. *et al.* Autism from 2 to 9 years of age. Arch. Gen. Psychiatry 63, 694–701 (2006). 16754843 10.1001/archpsyc.63.6.694

[b52] Lord C., Rutter M. & Le Couteur A. Autism Diagnostic Interview-Revised: a revised version of a diagnostic interview for caregivers of individuals with possible pervasive developmental disorders. J Autism Dev Disord 24, 659–685 (1994). 7814313 10.1007/BF02172145

[b53] Ashburner J. & Friston K. J. Voxel-Based Morphometry—The Methods. NeuroImage 11, 805–821 (2000). 10860804 10.1006/nimg.2000.0582

[b54] Ashburner J. & Friston K. J. Unified segmentation. NeuroImage 26, 839–851 (2005). 15955494 10.1016/j.neuroimage.2005.02.018

[b55] Ashburner J. A fast diffeomorphic image registration algorithm. NeuroImage 38, 95–113 (2007). 17761438 10.1016/j.neuroimage.2007.07.007

[b56] Philip R. C. M. *et al.* A systematic review and meta-analysis of the fMRI investigation of autism spectrum disorders. Neuroscience & Biobehavioral Reviews 36, 901–942 (2012). 22101112 10.1016/j.neubiorev.2011.10.008

[b57] Zielinski B. A. *et al.* Longitudinal changes in cortical thickness in autism and typical development. Brain 137, 1799–1812 (2014). 24755274 10.1093/brain/awu083 PMC4032101

[b58] American Psychiatric Association. The Diagnostic and Statistical Manual of Mental Disorders, Fifth Edition. (bookpointUS, 2013).

