# Supplementary material for: Anatomical imbalance between cortical networks in autism
Source: Sci Rep. 2016 Aug 3;6:31114. doi: 10.1038/srep31114 (PMC4971490; doi:10.1038/srep31114)
Supplement: Supplementary Information [file srep31114-s1.pdf]

Supplementary Information
for “Anatomical imbalance between cortical networks in autism”
by Watanabe and Rees

Supplementary Fig. 1

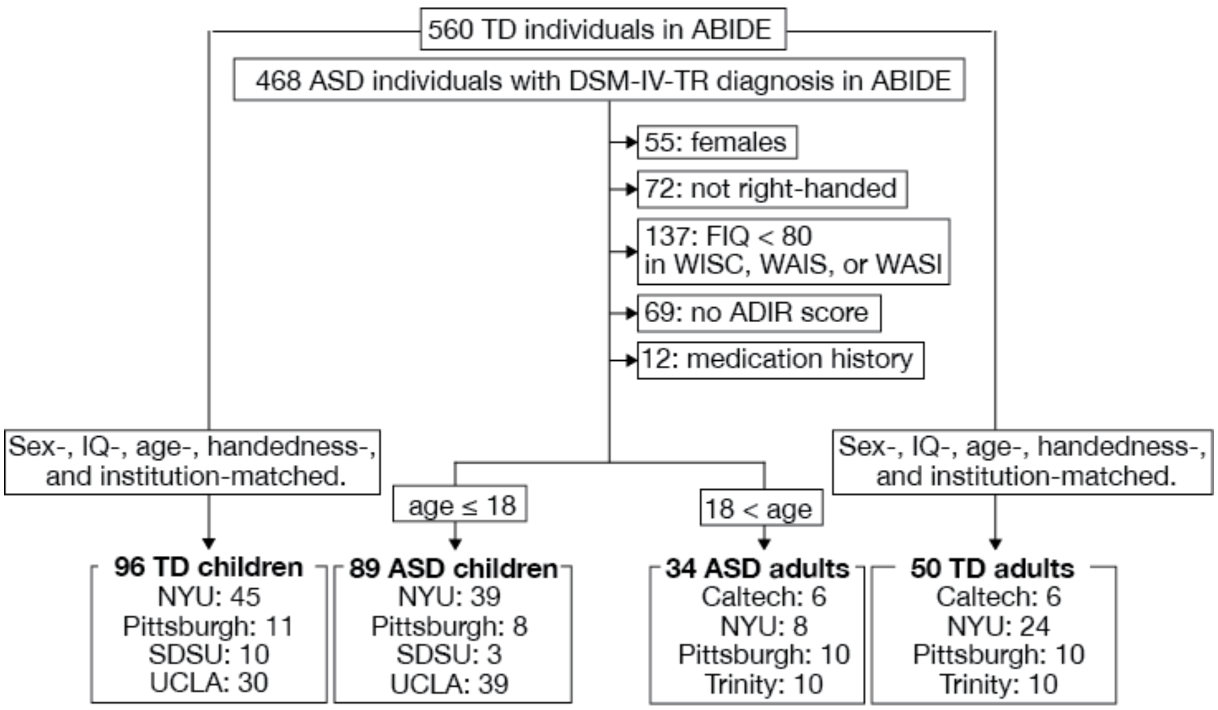

We used anatomical neuroimaging data shared in the Autism Brain Imaging Data Exchange. First, we selected data recorded from right-handed ASD males whose full IQ scores were ≥80 in a WISC, WAIS, or WASI test, and were given ADIR scores. Individuals who had medication history of anti-psychotic drugs or had medication on the scanning day were excluded. We divided these data into those of 89 ASD children with age ≤ 18, and those of 34 ASD adults with 18 < age.

We then selected data of sex-, IQ-, age-, handedness-, and institution-matched TD children and adults (see Table 1 for comparison of the demographic data). No significant difference in age and IQ was seen between different data collection institutes ($P \geq 0.1$; Supplementary Table 1).

UCLA, University of California Los Angels; NYU, New York University Langdon Medical Centre; SDSU, San Diego State University; Trinity, Trinity Centre for Health Sciences; Caltech, California Institute of Technology; ASD, autism spectrum disorder; TD, typically developing.

Supplementary Fig. 2

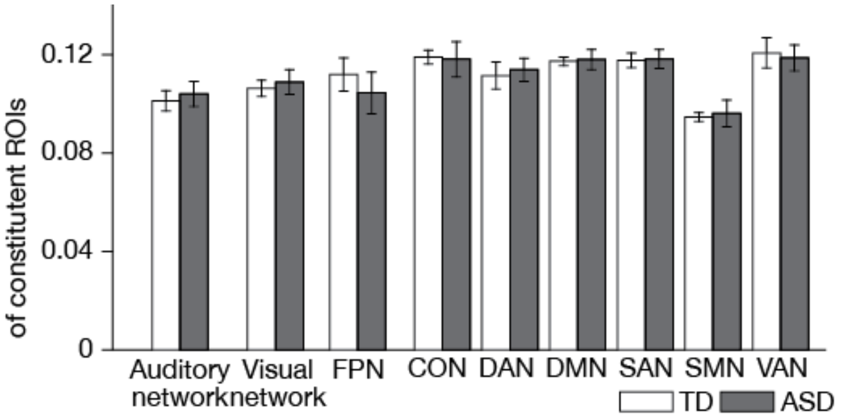

Comparison of network rGMVs in a relatively early part of childhood.
No significant difference in rGMVs between ASD and TD groups was found in any network in the relatively early childhood data (7≤ age ≤ 11; $P > 0.05$, in repeated measures two-way ANOVA). Error bars: std. The abbreviations of network names were spelled out in Fig. 1a.

Supplementary Fig. 3

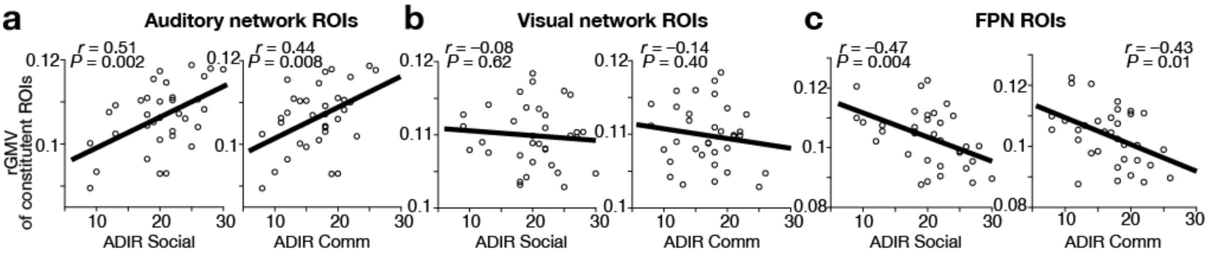

The correlations seen between rGMVs and severity of socio-communicational impairments (Fig. 4) were qualitatively reproduced even when the correlations were estimated separately using ADIR Social and ADIR Communication scores (Supplementary Table 4).

Supplementary Fig. 4

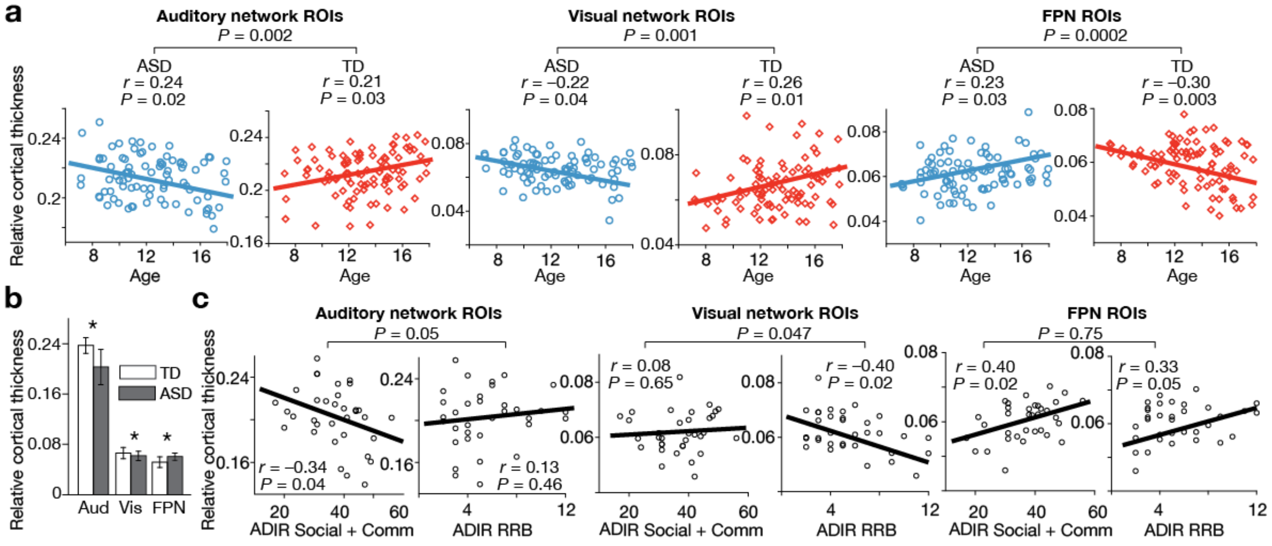

Results of cortical thickness analysis.

We repeated the analysis about anatomical imbalance between networks using preprocessed voxel-wise cortical thickness (CT) datasets shared in Preprocessed Connectomes Project (http://preprocessed-connectomes-project.github.io/abide/, Supplementary Methods).

Given that smaller CT values are relevant to more optimised and finer information processing[1,2], results of this cortical thickness analysis were qualitatively consistent with the original observations based on GMV.

a. Relative CT in auditory and visual network regions were conversely correlated with age in autistic children, whereas they showed positive correlations in TD children. The opposite pattern was observed in FPN regions. Although some of these correlations did not survive statistical corrections for multiple comparisons, these results suggest over-optimisation of information processing in auditory and visual networks in autistic individuals, and relatively less-optimisation of FPN functions.

b. Difference in relative CT between ASD and TD adults was consistent with such age-related changes seen in the data from children. In auditory and visual network regions, the relative CT values were significantly smaller in the ASD group than in the TD group, whereas the value of FPN regions was larger in the ASD group. * indicates $P_{\text{Bonferroni}} < 0.05$ in two-sample t-tests.

c. The atypical values of relative CT were correlated with autistic core symptoms. In auditory network regions, atypically smaller values of relative CT (i.e., over-optimisation of its function) indicated more severe socio-communicational symptom, whereas those in visual network regions were specifically related to severity of RRB symptom. In contrast, larger values of relative CT in FPN regions (i.e., less optimised information processing) were associated with severity of both core symptoms.

Supplementary Table 1

Comparison in age and full IQ between the different data collection sites

	ASD children		TD children		ASD adults		TD adults	
	F(3,85)	*P* value	*F*(3,92)	*P* value	*F*(3,30)	*P* value	*F*(3,46)	*P* value
Age	1.5	0.2	1.3	0.3	2.3	0.1	2.1	0.1
Full IQ	2.2	0.1	1.5	0.2	2.3	0.1	0.4	0.7

Supplementary Table 2

IQ-controlled age-rGMV partial correlations

		**Partial correlation between age and rGMV**			
		controlled by FIQ	controlled by VIQ	controlled by PIQ	ASD v TD
**Auditory network**					
	ASD children	0.31*	0.32*	0.30*	*P* < 0.05
	TD children	–0.054	–0.068	–0.056	
**Visual network**					
	ASD children	0.45*	0.44*	0.45*	*P* < 0.05
	TD children	–0.069	–0.076	–0.065	
**FPN**					
	ASD children	–0.32*	–0.33*	–0.30*	*P* < 0.05
	TD children	0.41*	0.44*	0.40*	

*, *P* < 0.05.

Supplementary Table 3

IQ/age-controlled symptom-rGMV partial correlations

	Partial correlation between ADIR scores and rGMV				
	controlled by FIQ	controlled by VIQ	controlled by PIQ	controlled by age	ADIR Social + Comm v ADIR RRB
**Auditory network**					
ADIR Social + Comm	0.48*	0.50*	0.47*	0.52*	$P < 0.05$
ADIR RRB	0.017	0.041	–0.004	0.06	
**Visual network**					
ADIR Social + Comm	–0.14	–0.15	–0.10	–0.12	$P < 0.05$
ADIR RRB	0.57*	0.55*	0.61*	0.56*	
**FPN**					
ADIR Social + Comm	–0.41*	–0.45*	–0.41*	–0.47*	ns
ADIR RRB	–0.37*	–0.39*	–0.36*	–0.43*	

*, $P < 0.05$.

Supplementary Table 4

Partial correlations between rGMV and social/communicational symptoms

	Partial correlation between ADIR scores and rGMV			
	controlled by FIQ	controlled by VIQ	controlled by PIQ	controlled by age
**Auditory network**				
ADIR Social	0.49*	0.50*	0.47*	0.52*
ADIR Comm	0.41*	0.49*	0.46*	0.44*
**Visual network**				
ADIR Social	–0.096	–0.097	–0.070	–0.12
ADIR Comm	–0.18	0.13	–0.066	–0.15
**FPN**				
ADIR Social	–0.43*	–0.46*	–0.41*	–0.46*
ADIR Comm	–0.34*	–0.39*	–0.38*	–0.43*

*, $P < 0.05$.

Supplementary Methods

Cortical thickness analysis

To validate our rGMV findings, we conducted the same analysis using preprocessed voxel-wise cortical thickness data recorded from the same individuals in Table 1. The preprocessed data were obtained from the ABIDE section in the Preprocessed Connectomes Project (http://preprocessed-connectomes-project.github.io/abide/). Among the 269 individuals in Table 1, cortical thickness data of four ASD children and two TD children were not found in the repository, and therefore, we analysed data of remaining 85 ASD children, 34 ASD adults, 94 TD children, and 50 TD adults. There was no significant difference in demographic data between ASD and TD groups ($P > 0.1$). According to the data repository, a cortical thickness value was calculated for each grey matter voxel by applying an automated preprocessing and analysis pipeline implemented in Advanced Normalization Tool[3] to a T1-weighted image of each individual.

To extract an average cortical thickness value for different anatomical structures, we first randomly divided a conventional grey matter parcellation map (here, AAL parcellation) to 1024 segments with similar numbers of continuous voxels using a random parcellation algorithm[4,5]. Next, we classified the grey matter segments into nine large-scale cortical brain networks (Fig. 1a): each segment was given one network label when ≥50% of the segment overlaps a specific network area which was defined as a collection of multiple 4mm-radius spheres around the network-specific coordinates[6,7]. Segments showing no sufficient overlap with any network were excluded in the following analyses.

By applying this grey-matter parcellation mask and network labelling to the voxel-wise cortical thickness data, we calculated the ratios of average cortical thickness values between the nine networks in the same manner used for calculation of rGMVs. We then performed the same analysis for these relative cortical thickness values as we did for the rGMVs.

Supplementary References

1. Song, C., Schwarzkopf, D. S., Kanai, R. & Rees, G. Neural population tuning links visual cortical anatomy to human visual perception. *Neuron* **85,** 641–656 (2015).
2. Jones, E. G. Microcolumns in the cerebral cortex. *Proc Natl Acad Sci USA* **97,** 5019–5021 (2000).
3. Tustison, N. J. *et al.* Large-scale evaluation of ANTs and FreeSurfer cortical thickness measurements. *NeuroImage* **99,** 166–179 (2014).
4. Zalesky, A. *et al.* Whole-brain anatomical networks: does the choice of nodes matter? *NeuroImage* **50,** 970–983 (2010).
5. Crossley, N. A. *et al.* The hubs of the human connectome are generally implicated in the anatomy of brain disorders. *Brain* **137,** 2382–2395 (2014).
6. Power, J. D. *et al.* Functional network organization of the human brain. *Neuron* **72,** 665–678 (2011).
7. Cole, M. W. *et al.* Multi-task connectivity reveals flexible hubs for adaptive task control. *Nat. Neurosci.* **16,** 1348–1355 (2013).
